# Chiral tunneling in gated inversion symmetric Weyl semimetal

**DOI:** 10.1038/srep21283

**Published:** 2016-02-18

**Authors:** Chunxu Bai, Yanling Yang, Kai Chang

**Affiliations:** 1 SKLSM, Institute of Semiconductors, Chinese Academy of Sciences, Beijing 100083, People’s Republic of China; 2 School of Materials Science and Engineering, University of Science and Technology Beijing, Beijing 100083, People’s Republic of China; 3 School of Physics, Anyang Normal University, Anyang 455000, People’s Republic of China

## Abstract

Based on the chirality-resolved transfer-matrix method, we evaluate the chiral transport tunneling through Weyl semimetal multi-barrier structures created by periodic gates. It is shown that, in sharp contrast to the cases of three dimensional normal semimetals, the tunneling coefficient as a function of incident angle shows a strong anisotropic behavior. Importantly, the tunneling coefficients display an interesting 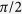 periodic oscillation as a function of the crystallographic angle of the structures. With the increasement of the barriers, the tunneling current shows a Fabry-Perot type interferences. For superlattice structures, the fancy miniband effect has been revealed. Our results show that the angular dependence of the first bandgap can be reduced into a Lorentz formula. The disorder suppresses the oscillation of the tunneling conductance, but would not affect its average amplitude. This is in sharp contrast to that in multi-barrier conventional semiconductor structures. Moreover, numerical results for the dependence of the angularly averaged conductance on the incident energy and the structure parameters are presented and contrasted with those in two dimensional relativistic materials. Our work suggests that the gated Weyl semimetal opens a possible new route to access to new type nanoelectronic device.

Recently, due to the abundant resemblances between high energy physics and condensed matter physics, Dirac materials, the materials host Dirac-like low-energy dispersions^1^, such as graphene^2^,^3^, topological insulator^4^,^5^, and Weyl semimetal^6^,^7^, have sparked a lot of interest in the physics community. Since the low-energy Dirac spectrum of the quasiparticles, these materials possess a lot of similar striking properties^8^. Some distinct features, however, are also revealed among them. In real space, unlike two dimensional (2D) graphene sheet, the Weyl semimetal is a three dimensional (3D) material. As for topological insulator, though it is a 3D system in general, the Dirac spectrum only hosts on each surface (3D topological insulators) or edge (quantum well structures). To some extent, they all can be regarded as 2D and one dimensional (1D) materials. In momentum space, in contrast to graphene and topological insulator, there is a 3D Dirac-like low-energy dispersions near nodal point in Weyl semimetal. Though the observation of Weyl semimetal was only experimentally achieved in 2015^9^,^10^,^11^,^12^,^13^,^14^,^15^,^16^,^17^, a lot of efforts have been made to hunt for the Weyl semimetal during the last five years^7^,^8^. For such a topological Weyl semimetal phase to occur, the materials must break time-reversal or inversion symmetry^18^,^19^. Up to now, it has proposed two kinds of simple electronic systems to realize Weyl semimetal in theory. One kind lacking time-reversal symmetry, includes pyrochlore iridates and magnetic topological insulator heterostructures^6^,^20^. The other kind obeys time-reversal symmetry and the candidates range from single crystal nonmagnetic materials to alloying^18^,^19^,^21^,^22^. Recently, following the theoretical prediction, the decisive signature for nontrivial topological Weyl semimetal and Fermi arc surface states are observed experimentally in nonmagnetic and non-centrosymmetric transition metal monoarsenides family by angle resolved photoemission spectroscopy^11^,^12^,^13^,^14^,^15^,^16^,^17^. Very recently, nontrivial topological properties in the proposed magnetic materials have also been confirmed by observing the key features (the Fermi arcs) in the material YbMnBi_2_^23^. Besides, the Weyl semimetal has also been predicted and subsequently realized in photonic crystals^24^. Those decisive experimental results, matching remarkably well with the theoretical results, confirm that the Weyl semimetal can be realized in condensed matter systems.

And for the both types above, the bulk band shows linear dispersions touching points (Weyl nodes) between the conduction and valence bands in the Brillouin zone and Fermi arcs excitations localized on the specific surface. Based on both the bulk and the surface states, a plethora of unusual and exotic transport features have been unveiled^7^,^8^. Specifically, the surface states known as Fermi arcs are believed to be responsible for novel quantum oscillations in magneto-transport and quantum interference effects in tunneling spectroscopy^7^,^25^. While, the bulk states are predicted to cause a large number of strange transport phenomena, such as negative magnetoresistance, quantum anomalous Hall effect, non-local transport and local non-conservation of ordinary current^7^,^26^ etc. In fact, those marvelous transport phenomena are associated with the chiral anomaly in Weyl semimetals which is absent in two dimensions. Because of the fundamental interest and the recent experimental success in Weyl semimetal^9^,^10^,^11^,^12^,^13^,^14^,^15^,^16^,^17^, it is crucial that further enthusiasm in the transport properties of the materials is aspired.

On the other hand, the research interest is partially fueled by the technological potential of the material in next generation analog and digital electronic devices exploiting the relativistic nature of the quasiparticals, such as ultra-high mobilities, the topological protection from back-scattering and the massless behavior of charge carriers^7^,^8^. Moreover, in the presence of electromagnetic fields, the bulk electronic states lead to the negative magnetoresistance, quantum anomalous Hall effect, non-local transport and local non-conservation of ordinary current, which also have pretty potential for device applications. To realize the device applications, controlling and understanding the transport properties of the designed electronic devices is a major goal in the physical and engineering sciences. Traditionally, the most powerful methods of manipulating a material’s transport properties rely on the typical tools, such as quantum confinement effect, doping, and superlattices. Among those tools, superlattices have provided an effective avenue for modulating the transport properties of semiconductor^27^, graphene^28^, and topological insulator^29^. Consequently, we may naturally envision the transport properties of Weyl semimetal should also be strongly affected by a superlattices structure. As one of the Dirac materials, Klein tunneling therefore is expected for Weyl fermion. Of special interest from the theoretical point of view, in contrast to a 2D Dirac-like band in graphene and topological insulator, Weyl semimetal is a 3D Dirac material and inherits a 3D Dirac-like band in momentum space. Thus, it is essential to study the chiral tunneling properties of Weyl fermions around the Weyl nodes in 3D Weyl semimetal and some intriguing features of the Klein tunneling effect between the 2D and 3D Dirac materials are expected. Moreover, while several groups have reported the exotic and unique properties in the specific structures with external electromagnetic fields^30^,^31^,^32^, the elucidation of the transport properties of electrostatic barriers in Weyl semimetal lacks. In view of the above, here we address the chiral tunneling properties by presenting an attempt at the theoretical evaluation of the transport properties of the massless Weyl fermions in the bulk state in the presence of single, double, and multiple symmetric electronic barriers since its importance both from basic point of interest and to Weyl semimetal electronic device applications.

The rest of the paper is organized as follows. In Section 2 we introduce a theoretical model and basic formalism used in the calculation. Then, Section 3 presents numerical results and a detailed analysis. In this section, the bulk state transport properties of a 3D Weyl semimetal encounter the single, double, and multiple symmetric electronic barriers are revealed. Moreover, the effect of the randomness on the probability of transmission and the conductance in superlattice structures of various sizes is studied. Finally, a short summary is given in Section 4.

## Model and basic formalism

**Figure 1:**
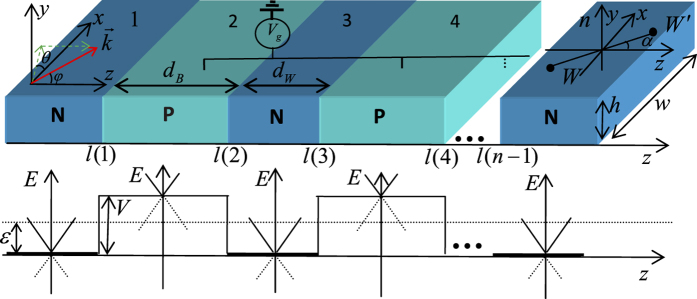
*Top*: Sketch of multiply potential barrier structures. The red arrow represents incident wavevector of the Weyl Fermions with angle 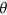 and 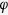. The black circles represent two Weyl points 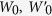 and the line 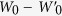 orients at an arbitrary angle 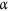 with respect to the z axis. 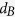 and 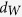 are the length of the potential barrier and potential well, respectively. *Bottom*: Illustration of the potential of the multiply potential barrier structures. The solid and dashed lines represent electron and hole bands, respectively.

**Figure 2:**
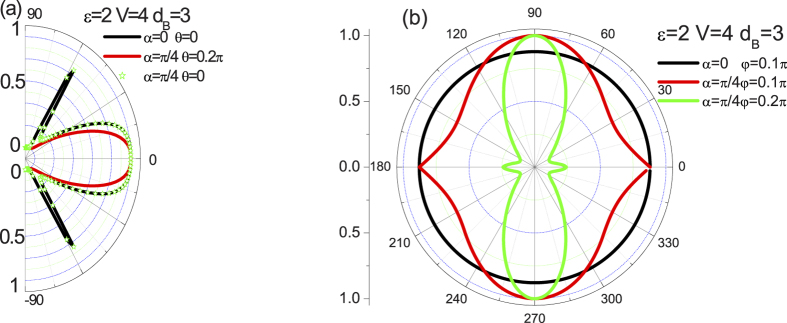
(**a**) 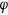-scan of the tunneling coefficients at 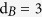, 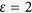, and 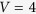. The black line, red line, and star correspond to the results for 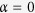
(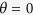), 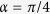
(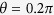), and 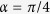
(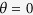), respectively. (**b**) 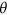-scan at 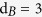, 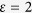, and 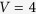. The black line, red line, and green line correspond to the results for 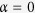
(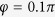), 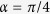
(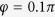), and 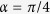
(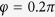), respectively.

**Figure 3:**
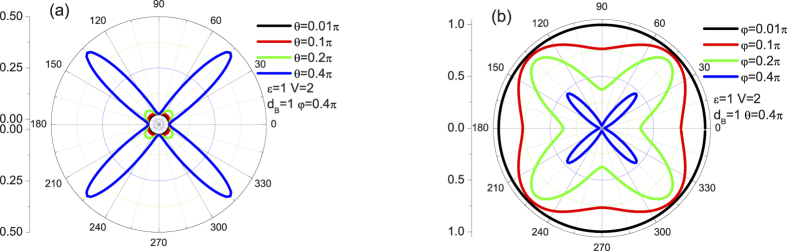
(**a**) 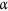-scan of the tunneling coefficients at 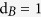, 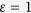, 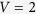, and 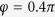. The black line, red line, green line, and blue line correspond to the results for 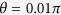, 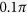, 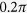 and 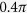, respectively. (**b**) 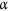-scan at 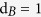, 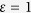, 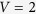, and 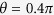. The black line, red line, green line, and blue line correspond to the results for 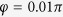, 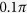, 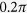 and 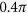, respectively.

In the present work, we consider an electron passing through a 3D inversion-symmetric Weyl semimetal-based structure with the spatially-modulated strength of electronic potential energy. The sketch of the structure and potential energy are shown in Fig. 1. The structure consists of two kinds of Weyl semimetal layer with different potentials, the first is a pristine electron-type Weyl semimetal without potential barrier (suppose it is zero) occuping the thickness 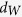, while the second is a hole-type part with potential barrier occuping the thickness 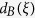 (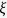 is the index with regard to barrier part), standing alternately. The spatial modulation of the strength of electronic potential energy can be realized by local chemical doping or by a top-gate lead sketched in Fig. 1. The growth direction is taken as the z axis, which is termed as the superlattice axis. In general, the axis connecting two Weyl points (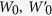), line 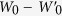, can be oriented at an arbitrary angle 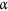 with respect to the normal of the potential barrier (the superlattice axis), after which one might envisage a Weyl fermion impinging on the interface from the angle (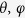)^33^. Note that the angle 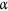 results in a rotation 
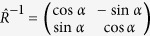
 operator which means that a vector is rotated around the y-axis by an angle 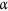
33. Such a similar rotation term is indeed present for graphene under uniaxial strain, but absent for an action on the pseudospins^34^. Due to the lack of the wavevector displacement, the pronounced asymmetric character in strained graphene vanishes (see Figs 2 and 3 for more details). The coordinate of the *i*th interface is marked by 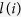. We focus here in the case where the height and width (along x and y direction) of the Weyl semimetal part, 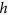 and 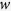, are much larger than 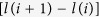 (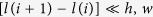). In this case the details of the microscopic description of the junction edges become irrelevant. Moreover, the disorder effect is taken into account in this way: the value of 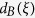 fluctuates around its mean value, given by 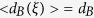 and 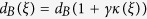, where 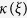 is a set of uncorrelated random variables 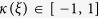. Here, the 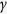 is the disorder strength.

The bottom subgraph in Fig. 1 shows the linear dispersion relation of the different parts for the electron and hole-type Weyl fermions, which are marked by solid line and dashed line. The electrostatic potential 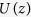 in Weyl semimetal-based structure may be adjusted independently by a gate voltage or by local chemical doping. In fact, recent experimental progress has been made in tuning the Fermi level in Dirac semimetal Cd_3_As_2_^35^,^36^. Specifically, via electrostatic doping by solid electrolyte gating, Liu *et al.* reported the observation of a gate-induced transition from band conduction to hopping conduction in Cd_3_As_2_ thin films last year^35^. Furthermore, by *in situ* alkaline metal doping, Liu *et al.* have also successfully tuned the position of the Fermi energy in crystallographic cell of Cd_3_As_2_^36^. As the similarities and potentially transition between Dirac and weyl semimetal phases, we can thus suppose an electrostatic potential will be induced in the weyl semimetal materials, which is similar to the production of Cd_3_As_2_ thin films based on the electrostatic doping by solid electrolyte gating^35^.

Due to the zero of potential is arbitrary, the potential profile of the system is the multiple quantum barrier structure which is given by


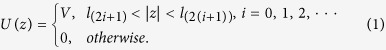


This is similar to the potential profile of conventional semiconductor, graphene, and topological insulator superlattice. The difference between them is that the charge carriers in the present structure are described by the following 3D Dirac-like Hamiltonian (a general anisotropic Weyl Hamiltonian) rather than the conventional Schrodinger Hamiltonian and 2D Dirac-like Hamiltonian^33^,^37^.


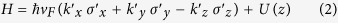


where 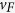 is the Fermi velocity, 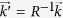 is the momentum measured from the weyl point, and 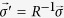 is the Pauli matrix of spins. The electron-like and hole-like quasiparticles in conventional semiconductor superlattice are generally described by separate Schrodinger equations with different effective masses, which are never interconnected in any way. In contrast, the electron-like and hole-like states in the graphene and topological insulator surface are interconnected, exhibiting chirality. They are described by two-component wavefunctions (spinor wavefunctions). Therefore, those 2D Dirac fermions with zero effective mass are qualitatively different from Schrodinger fermions. Here in 3D Weyl semimetal, unlike graphene and topological insulator, all three Pauli matrices are used in the momentum dependent Hamiltonian. The stable Weyl points (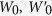) are topological objects in momentum space and always come in pairs with opposite chirality. It is thus natural to look forward to some novel phenomena in the study. In general, ideal linear dispersions are predicted (via angle-resolved photoemission spectroscopy and the first principal calculations) to range from 0.25 meV to 1 eV^11^,^12^,^13^,^14^,^15^,^16^,^17^,^18^,^19^,^38^ in various Weyl semimetal systems such as magnetic compounds BaYBi (Y = Au, Ag and Cu) and the family of nonmagnetic materials including TaAs, TaP, NbAs, and NbP. Physically, to avoid the inter-valley scattering (between 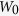 and 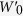), we must assume that the variation of the external periodic potential is much slower than the lattice constant (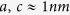)^12^,^13^,^15^,^16^,^17^. Meanwhile, we limit our discussion to the low-energy electronic states of Weyl fermion which have Fermi wavevectors close to the Weyl point based on the envelope function in the effective mass approximation. Those lead to a rectangular potential barrier for Weyl fermion in Weyl semimetal and the continuum Weyl Hamiltonian’s description is justified. In the light of the above statement, we thus start by considering the most general Hamiltonian as equation (2) describing a Weyl point.

In order to solve the transport problem in the Weyl semimetal superlattice (sketched in Fig. 1), we assume that the incident electron wave propagates at an angle 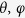. Following the ansatz in the form 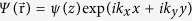 due to the translational invariance along the x and *y* direction, the general eigenstates of Eq.(2) can be obtained easily through a readily analytical derivation. Solving the eigenvalue equation 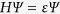, the eigenstates can be given as 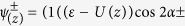

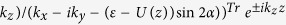
 where 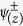 corresponds to quasiparticles moving along ± z directions and 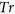 denotes the transpose of the row vector. In the study, we assume that the left and right parts of the structure are being pristine electron-type Weyl semimetal with a zero potential energy.

Let us now consider the case in which a Weyl fermion is incident from the left electrode. The wave functions in the left and right regions then read


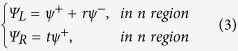


where 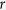 and 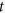 are the amplitudes of the normal reflections and transmission in the left and right electrodes, respectively. The wave functions in the middle regions are expressed as


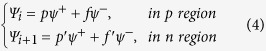


where 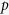 and 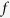 represent the reflections and transmission amplitudes in the barrier region, 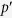 and 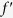 represent the reflections and transmission amplitudes in the pristine region.

Upon applying the continuity of the wave functions at the boundaries, the following transfer matrix is obtained:


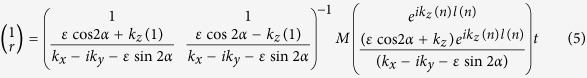


with 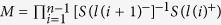, where 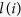 is the position of the interface and 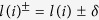 with infinitesimal positive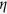, and 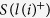 and 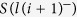 can be given by the z-dependent 2 × 2 matrixes, whose columns are constructed by the independent eigenstates of the Hamiltonian (2) as stated above. The transfer matrix can be expressed in general form as


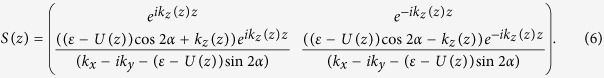


The position of interfaces 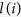 is straightforward given as 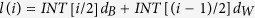 with i the layer number in the structure which is considered. While the z component of Fermi wavevector can be expressed in the form as 
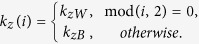
. Note that 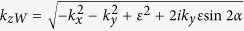 and 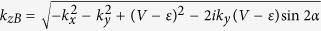 are the Fermi wavevectors out and in the barrier along the z axis. In particular, due to the translational invariance in the x-direction and the y-direction, the transverse momentum 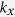 and 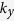
are conserved with or without a rotation with respect to the y-axis by the angle 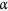. Moreover, by the present formula, the propagating and evanescent solutions are considered in the scattering process, which ensures the appropriate current conservation.

Then the angular dependence of transmission probability 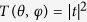 for such a structure can be obtained. If we take n = 3, it means a single barrier structure. To catch the principal physics behind the features of the chiral tunneling in Weyl semimetal, it is instructive to consider the case of 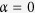. Then the transmission amplitude can be given in the following expressions:


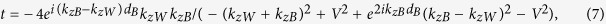


For the limit case (high barriers 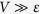), the above expression of t for the normal incidence 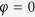 can be simplified into 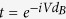. It means that the barrier remains always perfectly transparent for the normal incidence 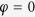. This is the most exotic feature termed as Klein paradox which is one of the direct consequences of the quantum electrodynamics^39^. Such intriguing phenomenon has been tested in condensed-matter physics: the strict 1D case in carbon nanotube^40^,^41^ and the 2D case of graphene^42^. Here the analysis of the Klein tunneling issue will be extended into the 3D case in Weyl semimetal.

For the double barriers case (n = 5), the above expression for t can be simplified as 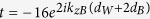

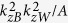
, (8)

with 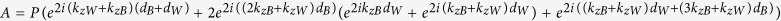 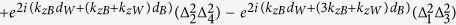 and 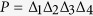. Where 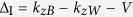, 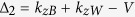, 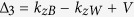, and 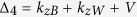. For the limit case (high barriers 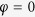 or 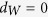), the above expression for t can be simplified into 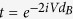. It means that the double barriers case equates to a single barrier case and a perfectly transparent can be achieved.

After the transmission coefficients are obtained, the zero-temperature tunneling conductance can be expressed by the integrating T over one-half of the Fermi surface^43^


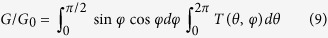


where 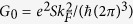 and 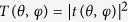. Here 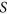 is the area of the junction in the x-y plane. Combining Eqs. (5), (6), and (9), the various conductivities for the Weyl semimetal-based structures can be obtained easily by the numerical calculations.

## Results and Discussion

### Transmission and conductance through a single potential barrier

#### Anisotropic transmission

First, we consider a single potential barrier structure and look for the effect of incidence angle 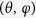, crystallographic angle 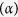, and structure parameters 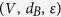 sign on the tunneling coefficients *T* and tunneling conductance 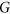. To generalize the calculation, we set all the quantities in the dimensionless units: 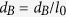, 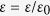, 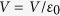, 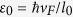 where 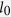 is the length unit. For 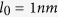, 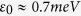. Throughout this study, quantities (In all of the calculations we used) corresponding to the same set of parameters are represented with the same line type. The intriguing tunneling characteristics of the transmission profile are presented in the Figs 2 and 5 as below.

Figure 2 shows the tunneling coefficients, *T*, of the incident electrons hitting a single potential barrier structure as a function of the angle 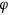 and 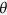 at 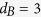, 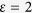, and 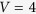. The other parameters are shown in the figure. Panel (a) shows the results as a function of angle 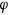 for a single barrier with different crystallographic angle 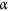 and incident angle 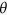. As expected, at normal incidence (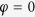), *T* = 1, irrespective of the values of other parameters. It is the feature unique to massless chiral fermions and directly related to the Klein tunneling. Although tunneling coefficients at normal incidence are not related to the crystallographic angle 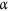
and incident angle 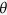, the angular 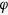 dependence of tunneling coefficients is. As we all know, lattice structure orientation is vital in determining materials’ fundamental properties. Due to high lattice symmetry, the graphene flakes exhibit isotropic behavior in general. However, the tunneling coefficient of weyl fermion, in sharp contrast to its counterpart in 2D graphene lakes, becomes anisotropic. It is clearly seen that the tunneling coefficient can be tuned for the case of 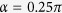 and 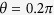. This indicates that quantum tunneling in the weyl semimetal becomes highly anisotropic due to the dimension and chiral nature of the quasiparticles, which is qualitatively different from the cases of 3D normal nonrelativistic and 2D relativistic electrons. Surprisingly, an intriguing situation that the crystallographic angle 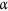 has no effect on the tunneling coefficient arises when we set incident angle 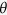 to zero or 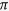. In effect, this can be intuitively understood from the fact that a 3D weyl fermion with 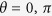 acts as an effective 2D relativistic quasiparticles which make the presence of isotropic behavior. That is to say, when we set 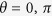, then our 3D tunneling issue reduces effectively to a 2D relativistic tunneling problem and the anisotropic disappears.

In order to show the effect of crystallographic angle 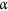 on the tunneling problem with respect to incident angle 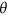 in such a junction, we have numerically calculated tunneling coefficient as a function of 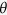 with different 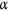 and 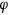 in Fig. 2(b). As explicitly shown, for 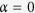, the tunneling spectrum exhibits an expected isotropic behavior, being nothing to do with the incident angle 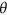 and 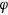 of the weyl fermion. While for 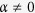, a clearly anisotropic behavior can be found. In particular, an increase of 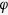 gives rise to enhancing anisotropic behavior. Such a with or without anisotropic behavior can be understood as follows. When 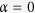, line 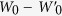 is along the z-axis and the wavevector in the left lead is 
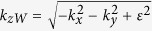
. It means that the wavevector along the z-axis keeps constant so that the tunneling spectrum is isotropic. As 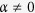, 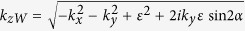 changes with the variation of 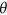, corresponding to an anisotropic behavior of the transmission. With the increase of 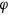, 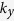 increases and the anisotropic behavior becomes more remarkable. Besides, a notable characteristic in Fig. 2(b) is the perfect tunneling at 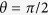 or 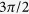 with 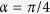, where the incident angle 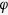 of the weyl fermion is nonzero. In effect, we have also testified that the perfect tunneling result can only be obtained at 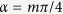 with 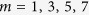, i.e., it gives rise to a 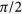 periodic oscillatory behavior. This result may be understood by the chirality of the weyl fermion. At 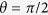
or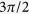 with 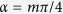, the matching between chiral quasiparticles inside and outside the barrier results in the perfect tunneling for any 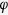. This is fundamentally different from the normal metal and the 2D relativistic cases.

The tunneling spectrum plots shown in Fig. 2 depend on the angle of incidence 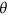 and 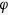 give rise to the anisotropic behavior at any angle except 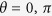 or 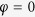. A more direct way to see the anisotropic behavior is shown in Fig. 3 where the tunneling spectrum is plotted as a function of the crystallographic angles 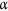 for constant 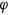 (a) and 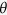 (b). As explicitly shown, the calculations reveal excellent agreement with the analysis above where a striking anisotropic behavior takes place for arbitrary 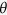 and 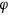 except some certain angles. Essentially, the maximum anisotropic behavior is reached for the crystallographic angles 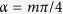 with 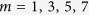, while disappears at 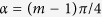. The periodicity 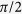 with 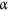 is easily seen. Moreover the angle-resolved anisotropic effect strengthens with increasing angle 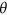 or 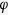 from 0 to 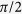. Those results suggest that both the lattice orientation and the incident angle can be used as the design variables to modulate device properties and optimize circuit performance in future integrated circuits based on Weyl semimetal materials.

#### Energy and structural parameters dependence of transmission

**Figure 4:**
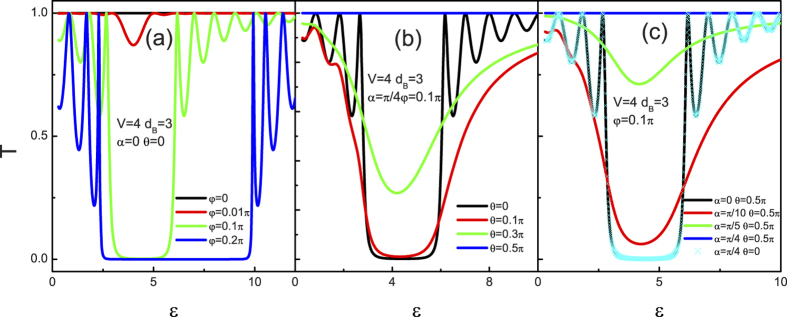
The dependence of tunneling coefficients on 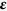 at 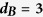 and 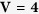. (**a**) for different 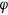 at 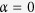 and 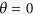. (**b**) for different 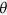 at 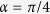 and 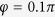. (**c**) for different 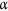 and 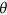 at 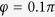. The other parameters are shown in the figure. Note that the results of 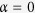 (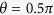) and 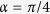 (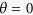) overlap with each other.

To gain a rough overview over the basic tunneling properties through the system, we also investigate the effect of incident energy and structural parameters. The calculated tunneling coefficients with different 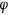, 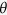, and 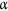 are plotted against 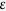 in Fig. 4(a–c) at 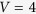 and 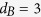, respectively. It is noted from Fig. 4(a) that an energy forbidden zone can be seen around 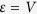 beyond certain value of 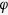 which means that the angle-dependent tunneling can be controlled by the incident energy of the weyl fermion. Moreover, it is clear that the tunneling coefficients are asymmetric with respect to the point 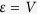 while an increase in the incident angle 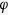 enhances the asymmetric effect and also the forbidden zone. Physically, the asymmetric effect attribute to different type tunneling process in the cases of 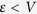 and 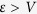. That is to say, a classical motion and a Klein tunneling, at least from the point of view of the transmission correspond to the case of 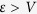 and 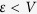, respectively. Meanwhile, the same oscillating features below and above the point 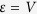 also stem from the distinct quasiparticle types. For energies lower than the barrier height, the tunneling resonances are due to hole-like quasiparticles inside the barrier through which the electron-like quasiparticles can tunnel. In contrast to the case of 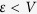, the quasiparticles through the structure origin from the conduct band when incident energies above the barrier height.

Figure 4(b) shows the tunneling coefficients of the single barrier junction as a function of 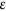 for various incident angle 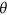 with 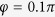 and 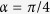. As shown in the figure, there are some same features as revealed above. First, for the case that when an incident angle 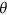 equates to zero or 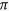, the tunneling properties through the structure would remain unchanged with respect to the crystallographic angle 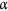. Second, the structure remains always perfectly transparent for 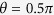 at 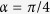, which is independent on the incident angle 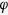 and the incident energy 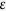. Besides, with increasing 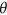, the energy forbidden dip slowly fades away and results in a perfect tunneling at last (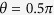). Here, it is also worth pointing out that, in contrast to the Klein tunneling case, the oscillating effect is more sensitive to the angle 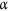. It is suggested that Klein tunneling is more robust than the classical tunneling with respect to the crystallographic angle.

To see the effect of crystallographic angle more clearly, the tunneling coefficients for various crystallographic angles 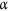 with 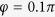 and 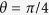 are shown in Fig. 4(c). It is clearly seen that, with increasing 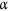, the energy forbidden dip slowly fades away and results in a perfect tunneling at last (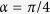) again. This indicates that the chiral tunneling nature of the weyl fermion plays an important role in the anisotropic tunneling, i.e., the the chirality of weyl fermions outside and inside the barrier does manifest itself only at any specifical angle (for example at 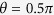 with 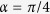).

**Figure 5:**
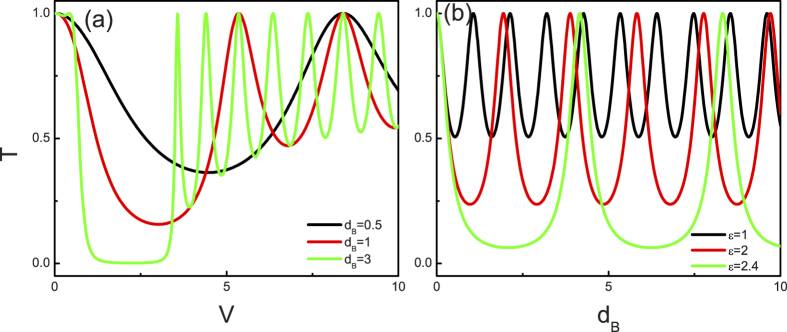
(**a**) The tunneling coefficients as a function of 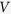 at 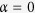, 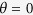, 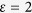, and 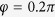. The black line, red line, and green line correspond to the results for 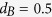, 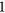 and 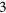, respectively. (**b**) The tunneling coefficients VS 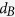 at 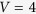, 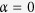, 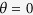, and 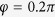. The black line, red line, and green line correspond to the results for 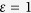, 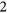, and 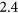, respectively.

Next, we present the results about the dependence of the tunneling properties on the structural parameters. Figure 5(a) shows the barrier height dependence of tunneling coefficients at different barrier widths for the present structure. Note that the tunneling coefficients for the single barrier structure oscillate with the barrier height. Comparing them with the case of short barrier (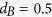), we find that more peaks appear with the increase of the width of the barrier. Furthermore, the feature of the tunneling dip is also related to the width of the barrier. In particular, the energy forbidden zone is only achieved beyond a certain value of 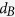. Figure 5(b) represents the corresponding results for the tunneling coefficients at different incident energy as a function of the barrier widths. There are again pronounced tunneling resonances at some barrier widths, where T approaches unity. The magnitude and period of the oscillation depend sensitively on the incident energy of the weyl fermion. With the increase of the incident energy (below the point 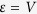), the magnitude and period of the oscillation become large. However, in contrast to Fig. 5(a) where the oscillation stems from electron-like quasiparticles in the barrier, the interface of the hole-like quasiparticles inside the barrier results in the pronounced tunneling resonances. Through the above analysis (in Figs 4 and 5), we recognize that the chiral tunneling in the gated inversion-symmetric Weyl semimetal junction can be tuned not only by the incident energy but also by the structural parameters.

**Figure 6:**
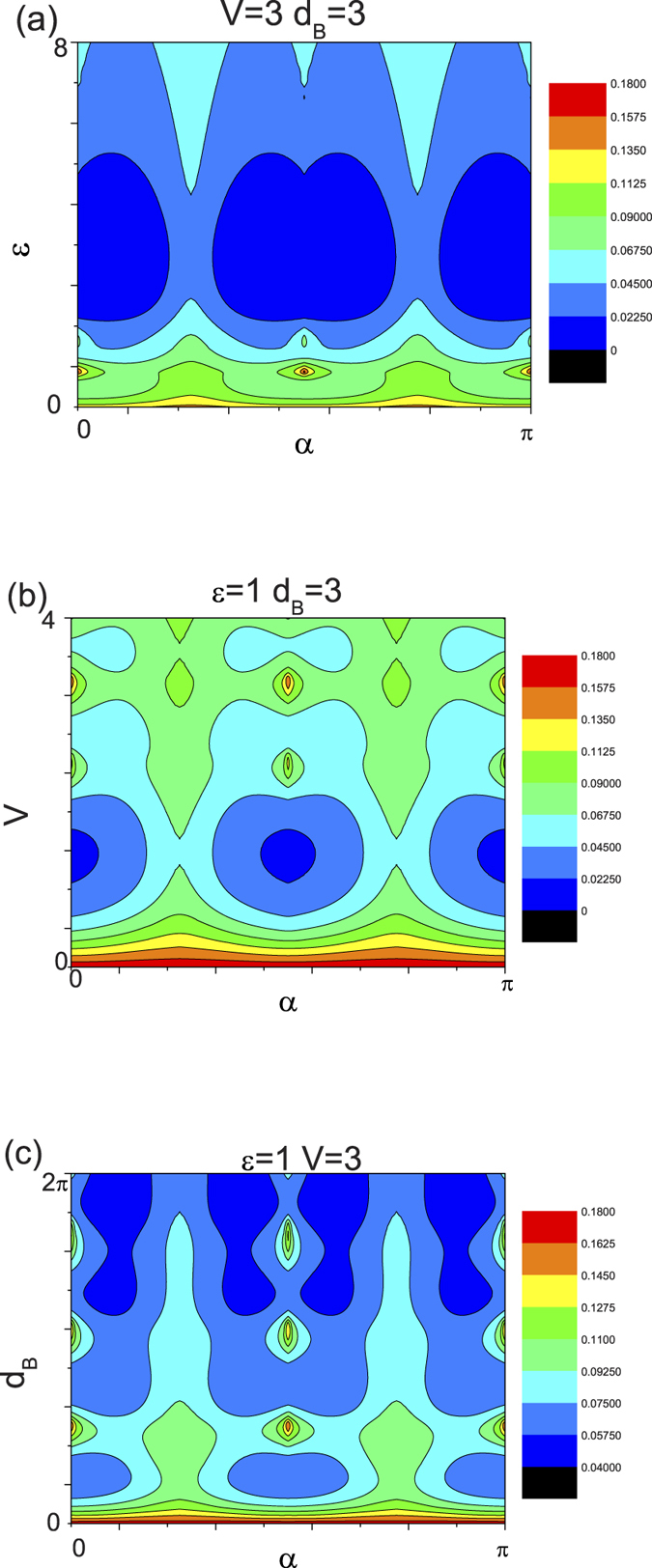
(**a**) The dependence of tunneling conductances on 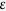 and 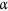 at 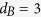 and 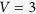. (**b**) The tunneling conductances as a function of 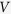 and 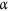 at 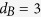 and 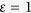. (**c**) The tunneling conductances *VS* 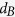 and 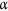 at 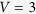 and 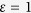. The oscillatory period 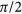 of the crystallographic angles 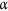 is clearly shown in the figure.

Such a property of the chiral tunneling leads directly to the situation that the 3D conductance is related to the incident energy and the structure parameters of the structure. In Fig. 6(a–c), we plot the dependence of 3D conductance for the single barrier structure on both the crystallographic angles 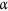 and the incident energy 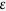, the barrier height 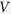, and the barrier width 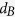, respectively. As shown in the figure, the 3D tunneling conductance represents sharp resonances with respect to 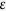, 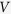, and 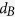 at a fixed 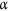. Therefore we can control the tunneling conductance by tuning the incident energy and the structure parameters. In fact, such a phenomenon corresponds to the Fabry–Perot like interference of electron-like or hole-like waves, which happens in the barrier region. Due to the incident energy and the barrier height can be effectively modulated by pure electrical method, it is suggested that the features of the tunneling conductance are vital to developing electrically controllable Weyl semimetal based device applications. More intriguingly, unlike 2D chiral tunneling of relativistic quasiparticle, the tunneling conductivity demonstrates a oscillatory behavior as a function of the crystallographic angles 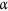 with a period 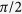. In particular, the resonance characteristic (about the incident energy 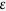, the barrier height 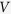, and the barrier width 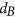) is very sensitive to the crystallographic angle 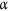, i.e., the maximum resonances behavior is reached for the crystallographic angles corresponding to 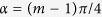 with 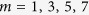, while it is less remarkable at 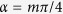. The features show a significant correspondence to the angular dependence chiral tunneling as elucidated above. It is worth to note that the maximum resonance behavior of the tunneling conductance corresponds to the case where the anisotropic behavior of the tunneling coefficients disappeares. This can be explained by the fact that the resonances depend strongly on the wavevector interference in the barrier region, a slight change in the value of the crystallographic angles 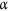, for a given situation, may result in a constructive and destructive modification of the resonance. Thus, the expected pattern of the chiral tunneling conductance in the Weyl semimetal materials also can be obtained by a suitable and ingenious structure (the crystallographic angle and the barrier width) design, which is more easily accessed in the 3D material in experiments.

#### Transmission and conductance through two potential barriers

Following the original suggestion of Tsu^44^, there has been a great deal of work on resonant tunneling in double barrier quantum well structures^45^. Most of the interesting phenomena in the semiconductor resonant tunneling diodes are based on Fabry-Perot type interferences arising from the impedance mismatch between the various layers. Recently, 2D relativistic like double barrier quantum well structure has been detailed discussion^28^,^29^,^46^,^47^,^48^. It is shown that the size of the well region plays a very important role in the tunneling of relativistic fermions via the obstacles created by the series of scattering potentials, because it associates with the bound states of the quasiparticles in well region and hence determines the criteria of the allowed resonance tunneling. Therefore, it is a natural question to ask what’s tunneling spectrum in a 3D Weyl semimetal based double barrier quantum well structures. This is not only due to its theoretical interest but also because such a structure could be used for building Weyl semimetal electronic circuits from appropriately engineer.

**Figure 7:**
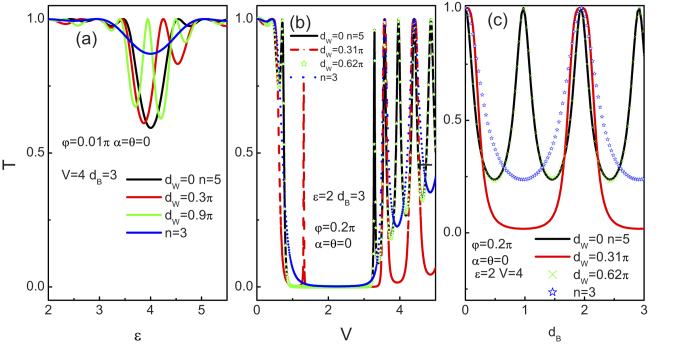
(**a**) The dependence of transmission coefficients on 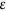 at 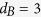, 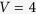, 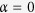, 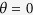, and 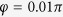. (**b**) The transmission coefficients as a function of 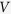 at 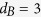, 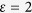, 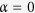, 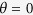, and 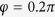. (**c**) The transmission coefficients VS 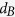 at 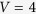, 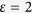, 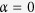, 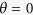, and 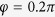. The length 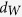 is shown in the figure and n = 3 means a single barrier structure.

In Fig. 7 we show the influence of the size of the well region 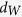 on the tunneling coefficient. As stated above, at normal incidence the tunneling coefficient *T* also does not depend on the structure parameters and the incident energy. Clearly, for a slightly diverged normal incidence (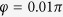) in Fig. 7(a), the structure remains always nearly perfectly transparent (except the energy zoon nearby the point 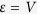 ), which is independent on the well width. Although the tunneling for a normal incidence is not related to the width of the well region, the oblique incidence is. Here, the quantum well region can be regarded as a cavity which can accommodate oscillating waves. Accordingly, the waves interfere in the well region gives rise to a Fabry-Perot like tunneling spectrum. The condition of such tunneling resonances is 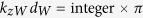. As 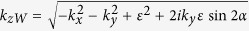, the resonance condition involves the energy, the length of the well, the incident angle and the crystallographic angles 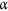. Indeed, for 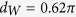 (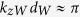) in Fig. 7(b,c), the tunneling spectrum reproduces the results of 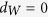. On the other hand if we cancel the well region by setting 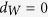, the resonance period may be twice smaller than that in a single barrier case. In effect, when the well width is zero, the present structures degenerate into the single barrier case, where the barrier width rises to twice in size (as compared to a single junction case). Thus the resonance strength doubles. From those results, we find that it is the distance 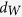 between the barriers that is important in determining the tunneling states and thus the tunneling spectrum. Therefore, the Weyl semimetal based double barrier quantum well structures can play a key role in the building Weyl semimetal electronic circuits.

### Superlattice

#### Angular dependence of bandgap

Comparing to the bulk material, superlattice or ordered arrays of metallic, insulating, or semiconducting solid, in general can be regarded as an artificial and an exciting new class of materials. Thus the superlattice structure has attracted a lot of attention since it is first brought forward in 1970^27^. Many unique electronics and opto-electronics properties such as fancy miniband effect, Wannier-Stark levels, and negative differential resistance are studied, which are essential for many applications^49^. Since one of the 2D relativistic material —graphene— is discovered in 2004, the transport properties of the 2D relativistic like superlattice are the most investigation focus due to its excellent and unique physical properties, e.g., the high mobility of carriers^8^. It is well-known that a plethora of intriguing characteristics in graphene with different superlattice patterns have aroused, such as, the new generation of massless Dirac Fermions and the highly anisotropic propagation^50^,^51^, the zero-k gap^52^, and the relation between the conductance oscillations and the bound states^53^. The similar chirality and dispersion properties between the Weyl fermion and the Dirac fermion in the graphene materials would enable the observations of those interesting phenomena which have been revealed in graphene. However, due to the dimensional difference between the 2D graphene and the 3D Weyl semimetal, we therefore can expect to observe some unusual features of the present 3D Weyl fermions. Most importantly, in 1D or 2D rectangular graphene superlattices, the calculations show that the transport properties of the 2D relativistic like superlattice depend closely on the superlattice structural parameters and the incident energy, even though the occurrence of Klein tunneling. It would, therefore, be worthwhile to survey how the transport properties of 3D Weyl semimetal based superlattice are modulated.

**Figure 8:**
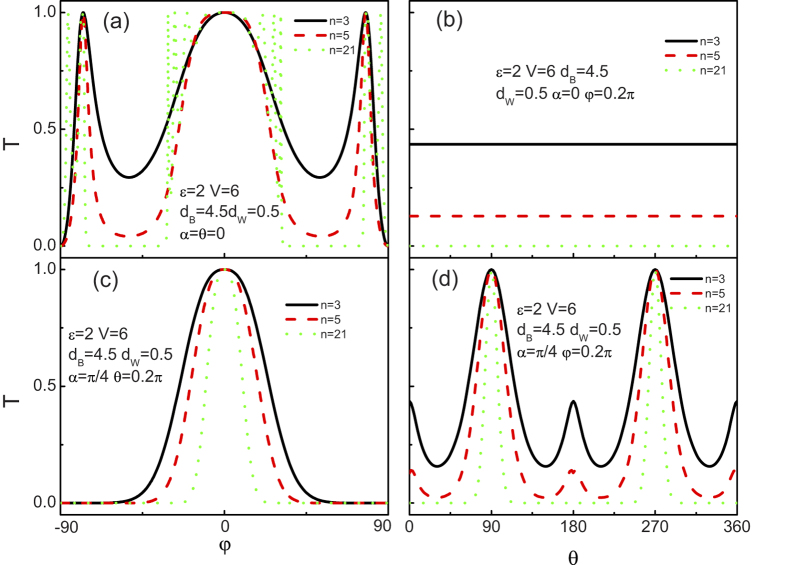
Tunneling coefficient T of chiral weyl fermion through 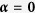 (a,b) and 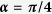 (c,d) as a function of the incident angle 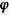 ((a) and (c)) and 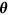 ((b) and (d)). The other parameters are shown in the figure.

Figure 8 shows the dependence of the tunneling coefficient on angular with the different number of the barrier. Figure 8(a,b) show the tunneling coefficient for the crystallographic angles 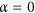 as a function of 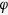 and 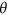, respectively. Figure 8(c,d) represent the corresponding results for 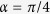. For 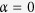, comparing them with the case of single barrier (n = 3), we find that more peaks appear for the case 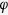 with the increase of the number of the barrier. Meanwhile, the tunneling dips of the present structure deepen with the increase of the number of the barrier and transform into the tunneling gaps when the number is big enough. While, the isotropic tunneling coefficient as a function of 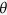 demonstrates a monotone attenuation feature with increasing the number of the barrier. Those indicate that the number of the barrier plays an important role in the tunneling in the present structure (even for the nonzero crystallographic angle as shown in (c) and (d)). Indeed, for 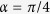, it is clearly seen from Fig. 8(c) that the perfect tunneling peak can only survive at the normal incidence angle (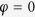), while the other two sharp peaks disappear. Moreover, the survival tunneling peak width shrinks with increasing the number of the barrier. As for 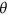, the tunneling processes of the chiral weyl fermion through such a superlattice are highly anisotropic in the case of 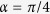. In particular, two tunneling peaks around 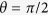 or 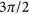 survive with increasing the number of the barrier. However, they are monotonously suppressed by the number of the barrier for the other angles and reduced to zero beyond a certain number of the barrier. In fact, such a perfect tunneling feature find a good agreement with analysis in the single barrier case (as shown in Fig. 2) and can be elucidated in a similar way. From these results, we conclude that the chiral tunneling in the Weyl semimetal material superlattice can be tuned by the number of the barrier due to the Fabry-Perot like interference of the chiral weyl fermion. Besides, the chiral tunneling in the present superlattice is highly anisotropic with respect to the crystallographic angles, which is qualitatively different from the case of 2D relativistic fermion.

**Figure 9:**
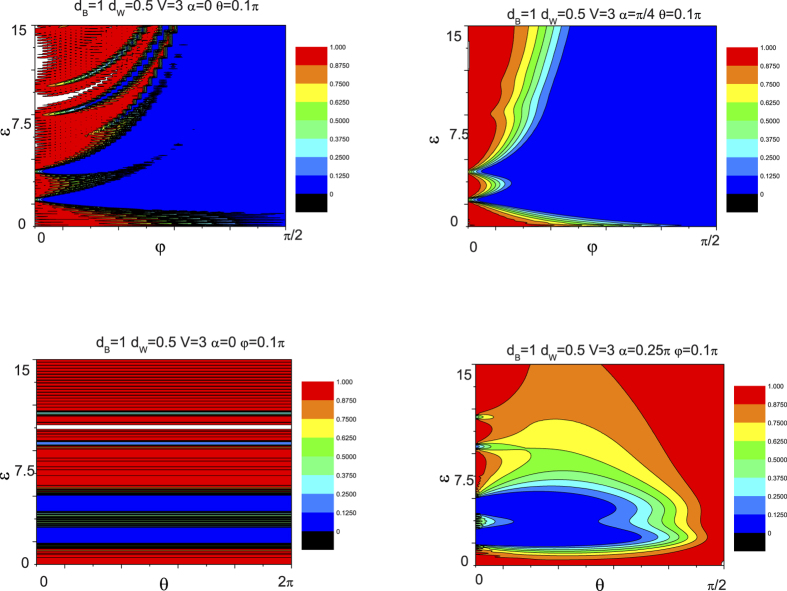
Tunneling coefficient T of chiral weyl fermion through 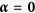 (a,c) and 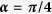 (b,d). Contour plots of tunneling coefficient T as a function of the incident energy 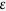 and angle 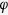 are shown in (a) and (c). Contour plots of tunneling coefficient T as a function of the incident energy 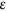 and angle 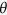 are shown in (b) and (d). The other parameters are shown in the figure.

In order to further understand miniband tunneling in Weyl semimetal based superlattice, the contour plots of the chiral tunneling as a function of both the energy (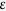) and angle (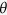, 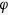) of the incident chiral weyl fermion are shown in Fig. 9. It is shown that the renascent minibands for the case of 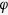 have a quarter circular form, similar to a whisker, and their widths decrease with increasing 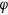 and virtually disappear beyond a certain angle. Besides, the resonances within minibands of this structure exhibit an increasing function of the number of the periodic arrangement of potential barriers or wells, which is not shown in the figure. Those features are basically similar to the conventional and graphene superlattices case. In turn, it is suggested that the well defined bandgaps irrespective of the angle of incidence (except normal incidence) can also possibly be obtained. Essentially the artificial engineering allowed and forbidden energy bands (well known as minibands and bandgaps) is a powerful technique for the design of new devices. For a nonzero 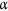
(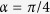), the results change a lot. Comparing to 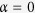, the bandgaps beyond the second one disappear and the critical angles of the minibands shrink to a small value. The phenomenon is more intriguing with respect to the incident angle 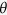 which does not exist in graphene superlattices. For the case of 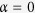, the isotropic tunneling coefficient as a function of 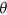 again demonstrates an isotropic miniband and bandgap structure. However, the isotropic characteristics (miniband and bandgap) are damaged by a nonzero 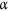. In contrast to the case of 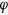, the bandgaps shrink and disappear at the certain value of critical angles. Meanwhile, there is clearly a wide domain around 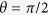 or 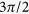 where the chiral tunneling can survive no matter what the value of the incident energy is. Note that the chiral tunneling gives rise to a 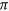-period function with 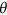, we only demonstrate the results as above shown. One more word, the miniband profile of a conventional superlattice can be described by a defined formula about the energy and momentum along the superlattice axis. For 2D relativistic fermion appropriate to graphene, Barbier *et al.* evaluate the dispersion relation in the presence of a 1D periodic potential^54^. It is shown that the dispersion relation in the y direction, in contrast to the nonrelativistic case, depends on 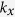 and is not the energy of a free particle. For the case of 3D relativistic fermion in Weyl semimetal, the situation is similar to that in the graphene case, but dispersion relation may becomes more intricate as the third component of the wave vector is involved. Besides, since the wave vector keeps a close relationship with 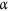, the dispersion relation in the present structure can be affected a lot by the crystallographic angles.

**Figure 10:**
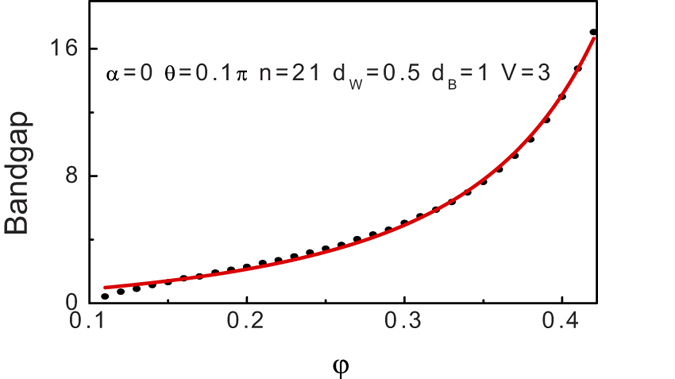
Angular dependence of the first bandgap in Weyl semimetal based superlattice. The solid-red line corresponds to the Lorentz like fit. In particular, the Adj. R-Square is 0.99774 for the Lorentz fit, indicating that the fitting is quite good. The other parameters are shown in the figure.

In order to gain a rough overview over the basic relationship between the incident angle and the bandgaps, we start with comparing the bandgaps with different incident angle. As we can see from the contours in Fig. 9, the first bandgap is more outstanding and robust than others. While it is too complex to give an analytical formula at the nonzero 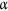 case, so we will focus on the first bandgap as a function of incident angle 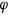 at 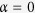. Excellent Lorentz fit was found, see solid-red line in Fig. 10. In particular, the Adj. R-Square is 0.99774 for the fit, which suggests that the fitting is perfect. It is worth to stress that Lorentz like relationship between the incident angle and the bandgaps is unique to this 3D Weyl semimetal based superlattice, since a parabolic dependence in a small angle and an exponential dependence at a big angle are found for the case of 2D relativistic fermion^55^. Besides, the first artificial bandgaps rang from 0.42 to 17 which have a strong appeal to the engineer community due to its multiply possible technological implications. Furthermore, the tremendous tunable energy range is a unique feature in Dirac materials as compare to the conventional meterials where the bandgap can not be changed unless replace the constituent materials. Experimentally, unlike the successful control of the light propagation in optics, the control of the incident angle is becoming one of the most challenging issue for electronics. Encouragingly, a sizeable advance in recent experimental has been made in order to reveal the angle-resolved contribution of electrons to the transport properties in two-dimensional materials, such as, graphene, transition metal dichalcogenides, and black phosphorous^56^,^57^,^58^,^59^. Specifically, the authors have been able to successfully distinguish the angle-resolved transport by using tilting metallic electrodes and angle-resolved electrodes. Similarly, we can expect that the unique angle-resolved nature in the 3D Weyl semimetal may be allowed for the realization by the current experimental technologies as those in 2D materials. Most importantly, due to its 3D structure, a higher efficient and more robust way to implement an angle-resolved measurement through the present structure can be envisaged as compared to the 2D materials.

#### Disorder effect

**Figure 11:**
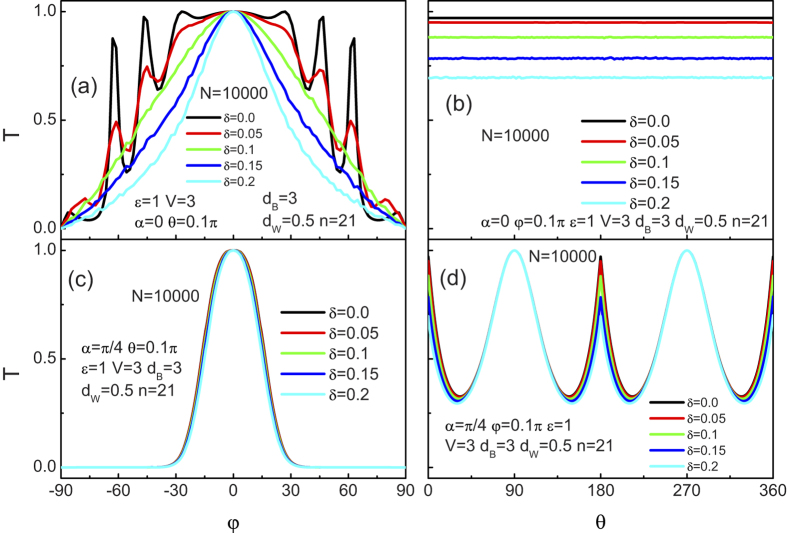
Tunneling coefficient T of chiral weyl fermion through the superlattice as a function of the incident angle 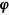 (a,c) and 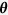 (b,d) for several disorder strengths at 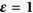, 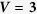, 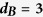, 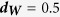, and n = 21. (**a**,**c**) with 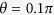. (**b**,**d**) with 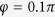. The disorder strengths are shown in the figure.

Since the unexpected transport properties of 2D relativistic structures can be brought about by the disorder, we provide a quantitative estimation about the effect of disorder on the chiral tunneling in this 3D Weyl semimetal based superlattice. Figure 11(a,b) show the tunneling coefficient for the crystallographic angle 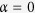 as a function of 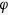 and 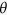, respectively. (c) and (d) represent the corresponding results for 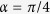. When 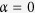 (see Fig. 11(a)), it is notable that the tunneling coefficient monotonically decreases with increasing the disorder strength 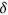 except the normal incident case 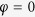. By and large, this is similar to the case in the 2D relativistic structure^60^. The feature of monotonic decrease stems from the destructive effect of the disorder on the interference effect. The resonance condition as given above depends closely on the product of the wave vectors of the quasiparticles and the structure parameters. Correspondingly, the resonance condition is destroyed by the disorder effect in the structure and the tunneling peaks monotonically decrease with 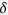. As analyzed in the section 2, the normal incident tunneling is robust against the disorder. While for a nonzero 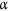
(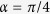), we can clearly see that the tunneling peak is independent on the 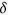 and the width of the tunneling peak becomes much smaller than the case of 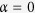. As for the case of 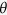, the apparent thing is that the isotropic tunneling coefficient can be also suppressed by the disorder effect. For a nonzero 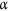
(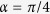), what is noteworthy else is that the tunneling peak keeps unchanged at 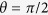 or 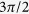, unlike the tunneling peak survival but decreases with increasing the disorder strength 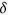 at 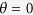 and 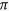. Once again this important novel feature can be found a good agreement with the investigation in Fig. 2. Especially, the above intriguing phenomena are completely lack in the 2D relativistic junctions since it is only a 2D material^60^.

**Figure 12:**
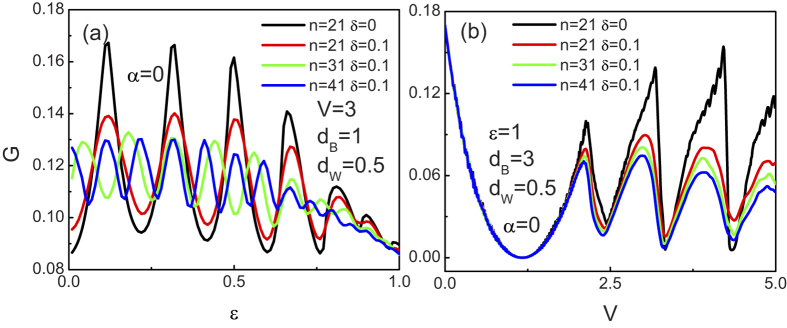
Tunneling conductance as a function of the incident energy 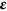 (a) and barrier height 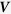 (b) at several different values of the number of the layer n and the disorder strength 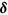. The parameters are shown in the figure.

Up to now, only the dependence of the tunneling coefficients in the superlattice on the disorder strength has been revealed. Since the conductance is more easily accessed in experiments than the tunneling coefficients, we now start to give a brief discuss about the angular averaged tunneling conductance. Figure 12 presents the tunneling conductance as a function of the incident energy 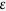 and barrier height 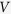 at several different values of the number of the layer n and the disorder strength 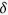. At n = 21 with different 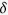, it is shown in Fig. 12(a) that the oscillate amplitude of the tunneling conductance decreases by increasing the strength of the disorder, while its average value almost keeps invariant. However, for the case of 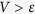, both the oscillate amplitude and the average value of the tunneling conductance decrease by increasing the strength of the disorder. At a constant strength of the disorder, changing the number of the layer n may also change the tunneling conductance, as depicted in Fig. 12. In general, the oscillate amplitude of the tunneling conductance in all cases decreases with increasing the system size. However, unlike the tunneling conductance as a function of 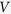 (as shown in Fig. 12(b)), the oscillate period of the tunneling conductance can be modulated largely by the system size. In effect, the resonance condition can be given by a function that relates to 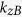, 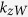, 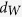, and 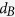. With the variation of n, the superlattice structure changes consequently which may change the resonance condition. Comparing to the case of 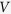, the wavevector both 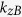 and 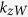 are tuned by the incident energy 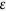 and give rise to a tunable oscillate period. These results are in complete contrast with those situations of a disordered conventional superlattice which becomes an insulator and a disordered 2D relativistic superlattice which holds a monotonical decrease feature until reaches a certain constant value in the thermodynamic limit. Here, the finding novel characters for the tunneling conductance of the superlattice should be important to the design of electronic nanodevices based on 3D Weyl semimetal materials.

## Conclusion

Based on the transfer-matrix method, we have investigated the chiral transport properties of the low-energy Weyl fermions in an array of the Weyl semimetal-based barriers structure created by applying the smooth scalar potentials, which cause no intervalley scattering. It is shown that, the direction of the crystallographic angles is always associated with the changes in the tunneling coefficients with respect to azimuth angle, or linked with the changes in the tunneling coefficients as a function of the elevation angle only for obliquely incident Weyl fermions, but do not affect the perfect normal tunneling process, no matter what crystallographic angle is. We will see that this behaviour is not maintained in the 3D normal nonrelativistic and the 2D relativistic materials. These features make the tunneling conductances of the Weyl fermions easily tunable by choosing the crystallographic angle.

For a double barriers structure, we mainly focus on the influence of the size of the well region on the tunneling coefficient. Clearly, the tunneling resonance states and thus the tunneling spectrum can be tunned by the Fabry-Perot type interferences between the potential barriers. Owing to those resonance features, such structure would be usefully used as building blocks in tunable electronic circuits.

We further study the fancy miniband transport properties and the disorder effect on the chiral tunneling through a superlattice. Our results show that the angle dependence of the first bandgap can be reduced into a Lorentz formula. Meanwhile, the possible opening bandgap exhibits a tremendous energy range by modulating the elevation angle. Importantly, we find a marked difference not only in the transmission but also in its angle averaged tunneling conductance as compared to the cases of a disordered conventional superlattice and a disordered 2D relativistic superlattice. That is to say, unlike the 2D relativistic case which provides a monotonously decrease feature until reaches a finite value, the mean value of the tunneling conductance almost keeps invariant with increasing the strength of the disorder. Furthermore, an exotic property also can be seen that, depending on the value of the incident energy, the disorder could either suppress or enhance the transmission. Therefore, controlling and tailoring the transport properties of such superlattice structures by the angle-dependent bandgap engineering and the disorder effect, at the applicant as well as at the theoretical level, hold considerable promise for the future practical electronic applications. In a word, we hope that the use of the scalar potentials will give more freedom to experimentalists to develop Weyl semimetal based nanodevices.

## Additional Information

**How to cite this article**: Bai, C. *et al.* Chiral tunneling in gated inversion symmetric Weyl semimetal. *Sci. Rep.* **6**, 21283; doi: 10.1038/srep21283 (2016).
